# A machine learning approach to predict perceptual decisions: an insight into face pareidolia

**DOI:** 10.1186/s40708-019-0094-5

**Published:** 2019-02-05

**Authors:** Kasturi Barik, Syed Naser Daimi, Rhiannon Jones, Joydeep Bhattacharya, Goutam Saha

**Affiliations:** 1 Department of Electronics and Electrical Communication Engineering, Indian Institute of Technology, Kharagpur, India; 2 Department of Psychology, University of Winchester, Winchester, UK; 3 Department of Psychology, Goldsmiths University of London, London, UK

**Keywords:** EEG, Prior expectation, Face pareidolia, Single-trial classification, Artificial neural network

## Abstract

The perception of an external stimulus not only depends upon the characteristics of the stimulus but is also influenced by the ongoing brain activity prior to its presentation. In this work, we directly tested whether spontaneous electrical brain activities in prestimulus period could predict perceptual outcome in face pareidolia (visualizing face in noise images) on a trial-by-trial basis. Participants were presented with only noise images but with the prior information that some faces would be hidden in these images, while their electrical brain activities were recorded; participants reported their perceptual decision, face or no-face, on each trial. Using differential hemispheric asymmetry features based on large-scale neural oscillations in a machine learning classifier, we demonstrated that prestimulus brain activities could achieve a classification accuracy, discriminating face from no-face perception, of 75% across trials. The time–frequency features representing hemispheric asymmetry yielded the best classification performance, and prestimulus alpha oscillations were found to be mostly involved in predicting perceptual decision. These findings suggest a mechanism of how prior expectations in the prestimulus period may affect post-stimulus decision making.

**Electronic supplementary material:**

The online version of this article (10.1186/s40708-019-0094-5) contains supplementary material, which is available to authorized users.

## Introduction

There is growing evidence that the ongoing brain activity is not meaningless, but rather carries a functional significance that largely determines how an incoming stimulus will be processed [1]. In other words, the conscious perception formed after the presentation of a stimulus could be causally shaped by the brain responses prior to the stimulus onset. In this framework, perception is understood as a process of inference, whereby sensory inputs are combined with prior knowledge [2], i.e., the integration of bottom-up sensory inputs and top-down prior expectations. To date, there has been no satisfactory functional explanation of the predictive role of prestimulus brain states. Although the role of prestimulus neural activity is unclear, it is found that perception is not entirely determined by the visual inputs, but it is intensely influenced by individual’s expectations, influencing the processing and interpretation of the stimulus on the basis of prior likelihood [3].

Earlier studies investigated the role of prestimulus event-related potentials (ERPs) on post-stimulus processing. For example, Mathewson et al. [4] revealed the influence of oscillatory microstates of cortical activity, manifested by alpha phase, on subsequent neural activity and visual awareness. In addition, both alpha power and larger fixation-locked ERPs are predictive of the detectability of masked visual targets. Fellinger et al. [5] found that prestimulus alpha phase is not randomly distributed in time across trials. Further, several neuroimaging studies employing visual stimuli demonstrated that the strength of prestimulus ongoing oscillatory activity, mainly in the alpha band, can indicate the future behavioral responses [6–9]. Here, behavioral responses often indicate whether a near-threshold stimulus will be perceived or not. Prestimulus brain states have also been shown to predict perceptual decisions [10–12] while resolving perceptual ambiguity to form a conscious percept for binocular rivalry stimuli [13–17]. Another study by Bode et al. [11] indicated that when stimuli provide discriminative information (pianos or chairs), decisions are predicted by neural activity following stimulus encoding, and when stimuli provide no discriminative information (pure noise), decision outcomes are predicted by neural activity preceding the stimulus. Furthermore, the sequence of preceding decisions (when stimuli contain discriminative information) biases the behavioral results of upcoming decisions in the case of pure noise stimuli.

In the current study, we extended this paradigm further, by using exclusively noise stimuli but informed participants that faces would be hidden in some of the noise images. This way, we emphasized the formation of expectation prior to the stimulus onset and investigated how prestimulus expectation would shape post-stimulus perception, seeing face or no-face, thereby removing the influence of stimuli with discriminative information on stimuli without such information.

The tendency of humans to perceive concrete (or familiar) images such as letters, animals or faces in random or unstructured noise stimuli is known as pareidolia. It is an extreme example of how prior expectation primes our perception. Face pareidolia is a psychological tendency to see faces in random stimuli. Among all forms of pareidolia, face pareidolia is more explored: Individuals have reported seeing a face in the clouds [18] or Jesus in toast [19]. We employed face pareidolia as an extreme example of the extent to which prior expectation can influence our perception. Face pareidolia indicates how the visual system is strongly predisposed to perceive faces, due to the societal importance of faces and our highly developed ability to process them. It also indicates inaccurate matches between internal depictions and neural inputs. Pareidolia is thus ideal for understanding how the brain integrates the bottom-up input of a visual stimulus and the top-down modulation of a goal-directed vision (e.g., to find a face in noise). Recent behavioral and functional imaging studies have provided some intriguing insights about how face pareidolia might emerge using a reverse correlation method [19–22]. These studies have demonstrated that the internal representation of faces underlying face pareidolia can be reconstructed experimentally based on behavioral responses. Hansen et al. [23], a similar method to reverse correlation was used to extract the internal representation of faces from brain activities measured by electroencephalography (EEG). These findings on face pareidolia suggest that the effect is not purely imaginary; instead, it has a neural basis. However, as the stimuli do not contain faces, face pareidolia clearly requires significant contributions of the brain’s interpretive power to detect and secure the vague face-like features to create a replica with an internal face representation. In this study, our principal aim was to investigate the role of prestimulus brain oscillations in predicting face pareidolia; hence, we strategically focused on the prestimulus period only (see [24, 25] for post-stimulus effect of face pareidolia) and performed single-trial classification employing machine learning framework using features extracted from the prestimulus brain oscillations.

While the perception of external sensory stimuli is a stimulus-dependent process, neuroimaging evidence of prestimulus activity suggests that it also depends on the brain states prior to the stimulus onset. However, decoding these brain states in terms of their functional roles is a complicated issue and critically depends on the behavior that is under investigation. In the current paradigm, we chose pure noise as the stimuli to investigate the causal relationship of prior expectation before the stimulus onset with individuals perceptions in face pareidolia. We estimated time-varying neuronal oscillations as features for our pattern classifier since large-scale brain oscillations observed spontaneously are critically associated with top-down processing that are predictive of future sensory events [26]. We performed classification at individual participant level. It was reported that the experimental designs that involve personalized model analysis require fewer subjects compared to those that involve subject-independent analysis [27]. Apart from the classification based on prestimulus activities, we also studied the temporal variations of our classifier’s performance in order to identify any critical time period before the stimulus onset. Additionally, we explored whether any specific brain oscillation plays a crucial role in predicting the perceptual decision. All analyses were performed at the single-trial level, thereby demonstrating the usefulness of machine learning techniques in decoding mental states from prior brain states [28–30].

## Materials and methods

### Participants

Seven healthy human adults (6 females, age range $23.43 \pm 4.20$ years) participated in this study. All participants were neurologically healthy, not taking any medication at the time of experiment, and had no history of mental disorders. All participants gave written informed consent prior to the experiment. The experimental protocol was approved by the Local Ethics Committee at Goldsmiths, University of London.

### Stimuli

**Fig. 1:**
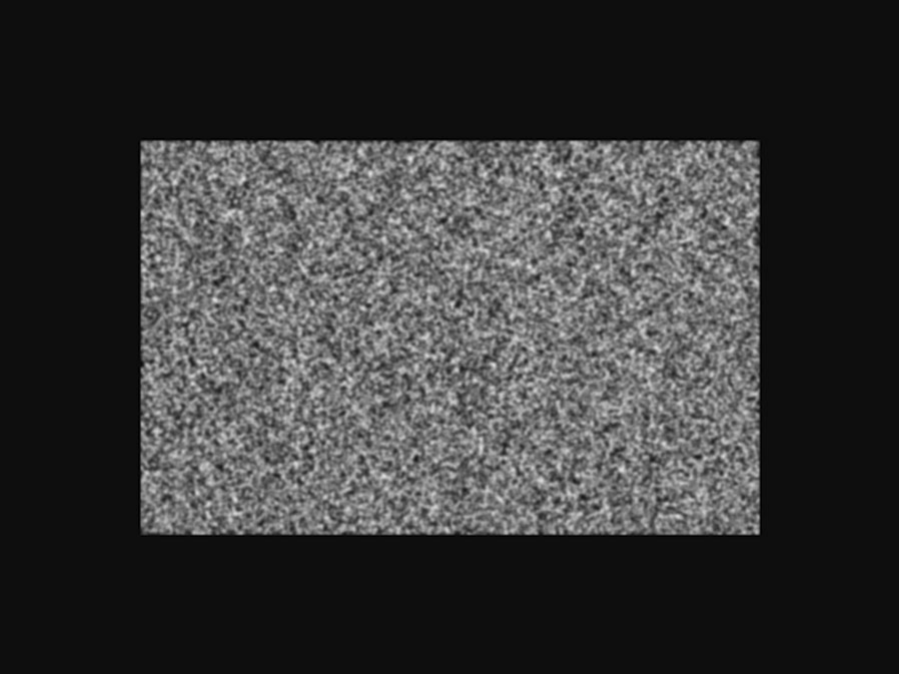
An example of visual noise image that was classified as ‘face’ by the six out of seven participants

In our experiment, visual white noise stimuli were used. The images were generated using Adobe Photoshop V.9^®^. A total of 402 images were used, which were slightly different from each other. However, these images were made to the same specifications. These were rectangular images on a black background, with monochromatic noise and a 100% Gaussian distribution, and had a Gaussian blur with 1 pixel radius. One example image that was classified as ‘face’ by the six out of seven participants is shown in Fig. 1.

### Procedure

The experiment was composed of six blocks, each separated by 2-min rest breaks. Each block contained 67 trials. In each trial, a central fixation cross was presented for 1000 ms, followed by the visual noise stimulus presented centrally, for 369 ms. A screen then appeared asking participants whether they had seen a face, to which participants responded with an appropriate button press to indicate their response. Jitter was introduced in between trials. Stimulus presentation and responses were controlled by the E-prime^®^ (Psychology Software Tools, Inc., USA).

Before beginning the task, participants were informed that faces had been hidden in some of the images; however, only noise images were used throughout. Participants were instructed to keep concentrating as the duration of the image presentation was short.

### Data acquisition and preprocessing

**Fig. 2:**
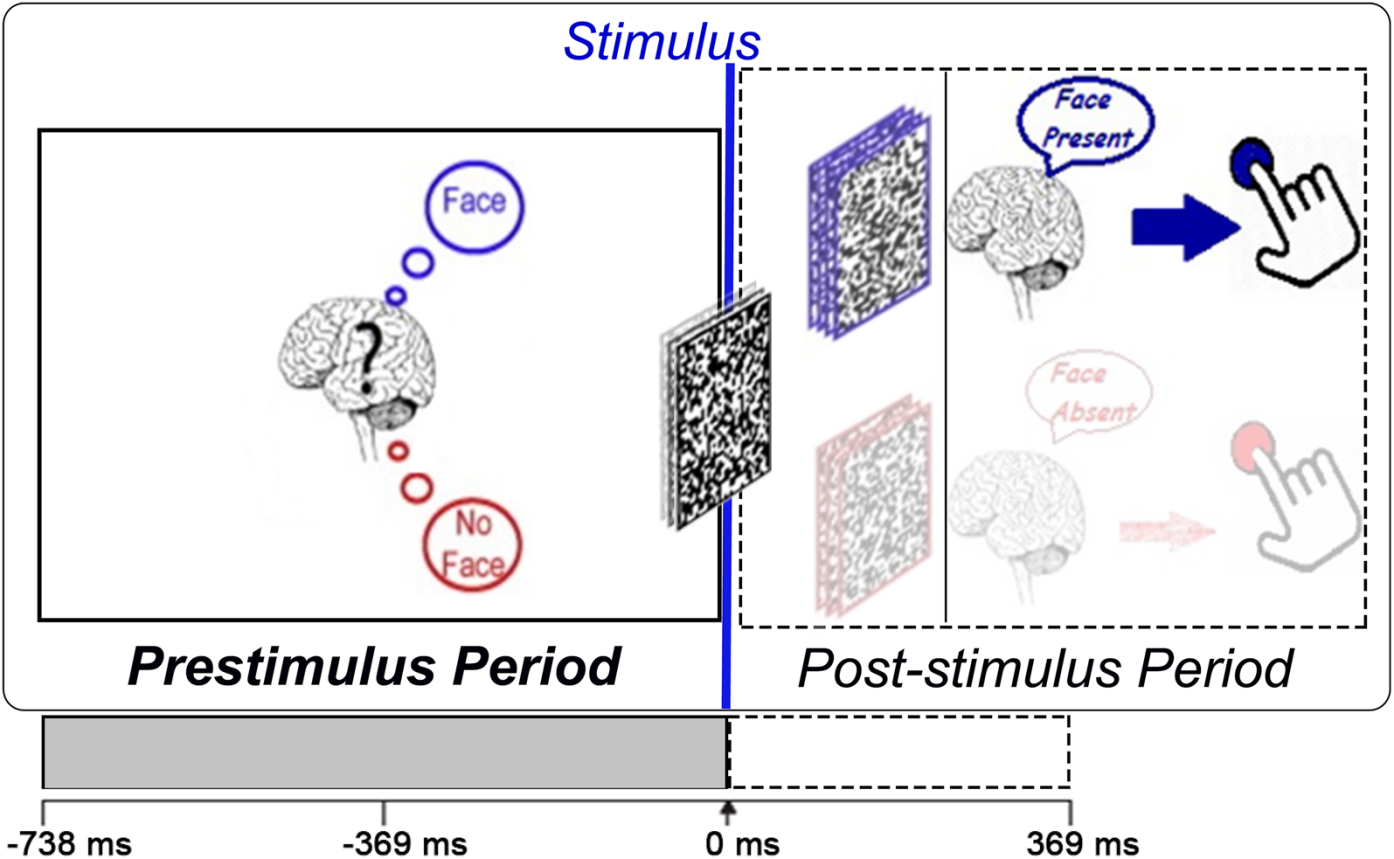
Experimental paradigm: stimuli were randomly produced visual white noise images. To influence participants’ prior expectation, they were informed that in some of the trials, face would be hidden in the noise stimulus. After stimulus onset, participants were instructed to press one of the two buttons to indicate whether they perceived a face or not. Here, an example of an epoch ($-\,738$ ms to 369 ms) is presented. Time *t = *0 represents the stimulus onset. In this study, we focused the 738-ms time period (represented in gray) before the stimulus onset

EEG signals were acquired using 64 active electrodes placed according to the international 10–10 system of electrode placement. The vertical and horizontal eye movements were recorded by placing additional electrodes above and below right eye and at the outer canthus of each eye, respectively. The EEG signals were amplified by BioSemi Active Two amplifiers and filtered between 0.6 and 100 Hz. The sampling rate was 512 Hz. The EEG data were algebraically re-referenced to the average of two earlobes. We applied notch filter at 50 Hz to reduce any powerline interferences. Blink-related artifacts were corrected using independent component analysis (ICA). Further, any epochs containing large artifacts were rejected based on visual inspection. In this study, as we focused on investigating the predictive power of the prestimulus brain responses, we epoched our data from 738 ms before the presentation of an image to 369 ms following the presentation. The experimental paradigm and the epoch formation are shown in Fig. 2.

Each trial was categorized as one of the two classes, *Face* class or *No-face* class, depending on participants response on trial-by-trial basis. The number of trials in each class for individual participant is listed in Table 1. The EEG data were preprocessed and analyzed by MATLAB-based toolboxes, EEGLAB [31] and FieldTrip [32], and by custom-made MATLAB scripts.

**Table 1:** Number of trials of each subject

Subject	No. of trials present in face class	No. of trials present in no-face class
Subject1	67	193
Subject2	68	226
Subject3	116	212
Subject4	104	187
Subject5	90	116
Subject6	116	216
Subject7	159	170

### Feature extraction

Wavelet-based time–frequency analysis is widely used in brain signal studies [33, 34]. We used complex Morlet wavelet with four cycles. A short wavelet with few cycles has a better time resolution than a wider wavelet with more cycles [27]. Each EEG signal was decomposed into frequency components from 1 to 40 Hz in steps of 1 Hz [35], producing the time–frequency power spectrum (TFPS). Next, we calculated frequency band-specific spectral power in classical EEG frequency bands as follows: delta (1–4 Hz), theta (4–8 Hz), alpha (8–13 Hz), beta (13–30 Hz) and gamma (30–40 Hz). The prestimulus period was divided into short 10-ms segments without overlap, resulting in 74 segments, and the mean spectral power of each 10-ms segment was subsequently computed. Therefore, for every channel/trial/participant, we obtained 5 (frequency bands) $\times$ 74 (segments) = 370 features. Further, we derived the various feature sets as follows.

- TFPS features were extracted from all electrodes, and the feature dimension was 23,680 [electrode (64) $\times$ frequency band (5) $\times$ time window (74)]. This feature type was named as TFPS64 (time–frequency power spectrum of 64 electrodes).
- Next, the time–frequency power spectrum for each hemisphere was acquired [36]. We had 27 electrodes located in each hemisphere, and this feature was named as TFPSL (left) or TFPSR (right).
- Next, we computed the cerebral asymmetry by calculating the difference between the time–frequency power spectrum of two cerebral hemisphere (left–right). It was labeled as DATFPS (differential asymmetry of TFPS). The asymmetry indices were calculated at each of the 27 electrodes by power subtraction (e.g., TFPS of Fp1–TFPS of Fp2). For each of TFPSL, TFPSR and DATFPS feature types, we had 9990 features [electrode (27) $\times$ frequency band (5) $\times$ time window (74)].

**Fig. 3:**
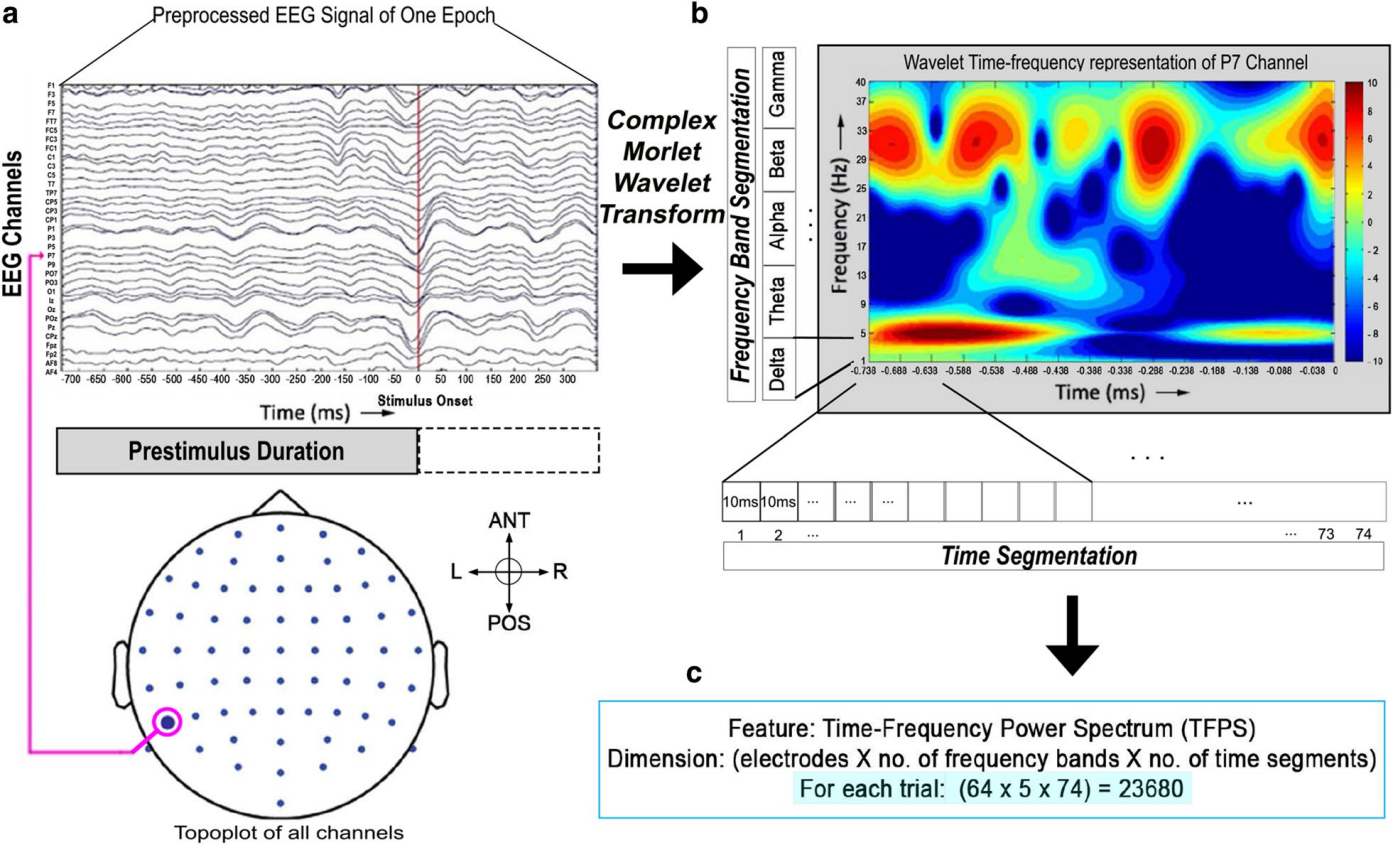
Feature extraction procedure: **a** A typical epoch of EEG channels. Red vertical line denotes stimulus onset. **b** Time–frequency representation (TFR) of one EEG channel (here P7, chosen randomly) obtained by convoluting the EEG signal with complex Morlet wavelet. The prestimulus period was segmented into nonoverlapping 74 short windows of 10 ms each. Similarly, frequency band segmentation also produced five segments by band-wise averaging of each frequency point within individual frequency band (see *Materials and methods*). **c** Feature dimension of time–frequency power spectrum (TFPS) that was extracted from all 64 EEG electrodes

Figure 3 clarifies each step of feature extraction procedure.

### Feature selection

Before performing feature classification, feature selection is an important preprocessing step in machine learning. The objective of feature selection is to extract a subset of features by removing redundant features as well as keeping the most relevant features [37, 38]. It is effective in dimensionality reduction, eliminating irrelevant features, improving learning accuracy and increasing result comprehensibility. We used the Student *t* test for feature selection because it performs better than the complex wrapper and embedded methods, especially when there are a large number of features [39]. It is to be noted that the relevance ranking methods (e.g., *t* test) take relatively less computation time [40] for feature selection.

As our primary goal here was to reduce feature dimension but not interpret their statistical significance, multiple comparison problem was considered not relevant [41], and therefore, we used uncorrected *p* values to rank the features. From ranked features, we selected a subset of the features that were below the chosen *p* value thresholds. These thresholds were only used to obtain a coarse selection of features in order to reduce the feature dimension. Different thresholds were employed to investigate the effect of increasing the number of selected features [42]. We do not interpret the relative relevance of the selected set of features according to their *p* values, rather making them equal members of a larger pool to formulate a classification model that puts its own weight against each feature [39, 43, 44].

### Single-trial classification

As stated earlier, we had two classes of trials depending on the participant’s responses: *face* and *no-face*. Our classifier, based on the prestimulus EEG data, aimed to categorize each trial to one of these two classes. We considered personalized average model (PAM) where trials of individual participants were handled independently for studying participant-dependent characteristics [45].

**Fig. 4:**
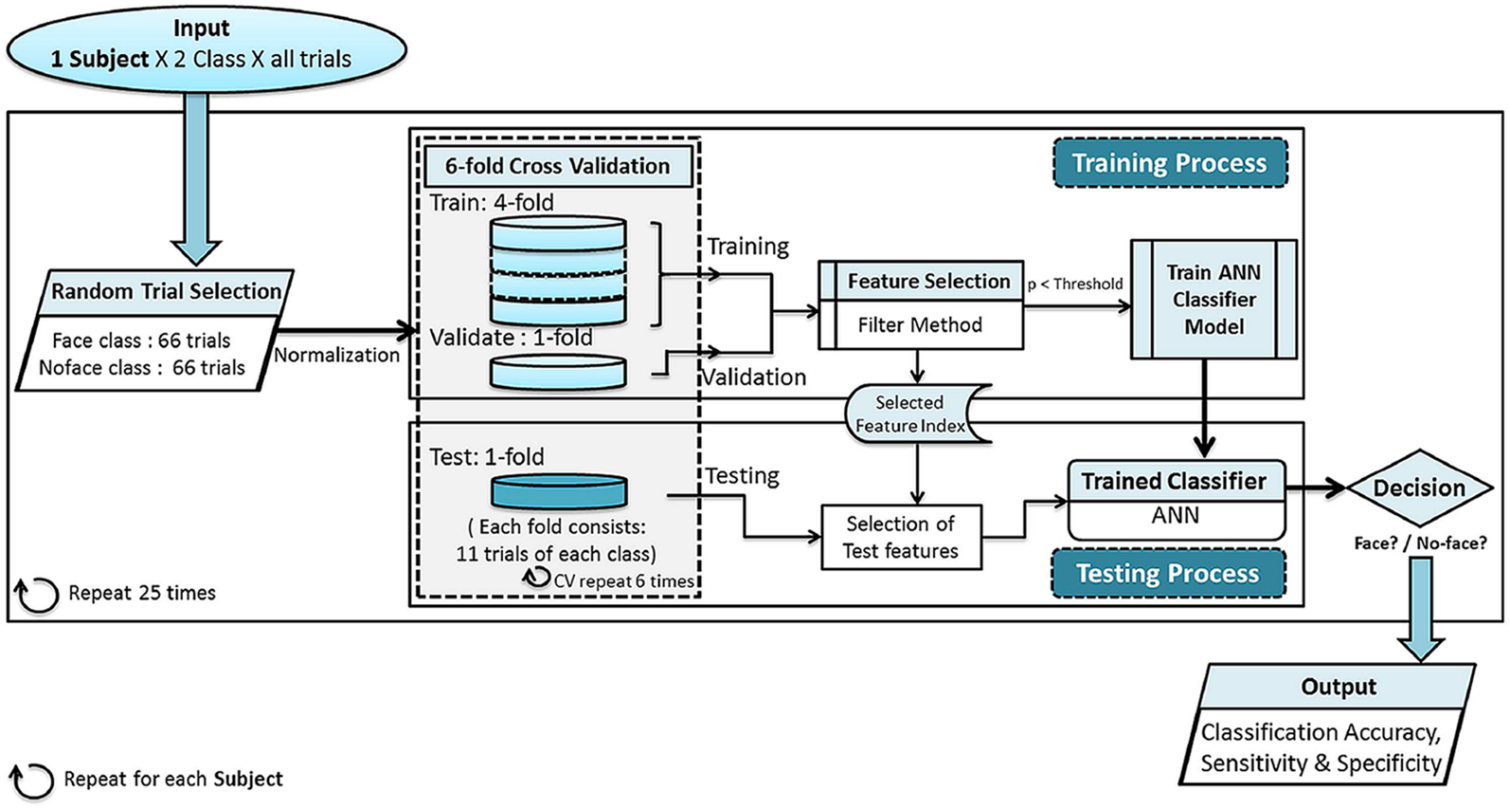
Block diagram of classification process for personalized average model: all trials of each subject were proceeded to the main classification block. Random downsampling was performed to remove data imbalance from *face* and *no-face* classes. Then typical machine learning classification process was executed with sixfold nested cross-validation technique. Here simple filter feature selection technique (*t* test) was followed by artificial neural network for the two class problem. Finally, the outcomes are classification accuracy, sensitivity and specificity of each subject

The number of trials in the *no-face* class was much higher than that in the *face* class (Table 1). To overcome the class imbalance, we used random downsampling approach [46, 47]. In this method, the majority class was randomly downsampled to equate the number of minority and majority class samples, ensuring the balance between two classes. Here, 66 trials were used from each class. Since this method used only a subset of majority class samples, the data were rotated 25 times to minimize selection bias; see Fig. 4 for block diagram of the detailed classification process.

We used artificial neural network (ANN) [48, 49] as a classifier with sixfold nested cross-validation (CV). The two-layered feedforward back-propagation ANN consisted of an input layer, a hidden layer of 10 neurons and an output layer with two neurons representing the two classes. The number of neurons in the input layer changed according to the feature type and number of features selected. The neural network was trained using scaled conjugate gradient back-propagation algorithm [50]. In ANN, the maximum number of cycles was allocated as 10,000 and the mean squared error or the performance goal was set to 10e${^{-5}}$. The hyperbolic tangent sigmoid transfer function was used as the activation function. Prior to classification, the feature vectors were normalized between 0 and 1. To prevent the overfitting of the ANN classifier, early stopping of training using validation set was employed. In each fold of CV, the available data were divided into three subsets. The first subset was the training set, which was used for computing the gradient and updating the network weights and biases. The second subset was the validation set. The error on the validation set was monitored during the training process. The validation error normally decreased during the initial phase of training, as did the training set error. However, when the network began to overfit the data, the error on the validation set typically began to rise. When the validation error increased for a specified number of iterations, the training was stopped, and model for minimum validation error was returned. The sixfold nested CV was performed with different randomly selected datasets of a participant to address data imbalance. To increase reliability, this procedure was performed 25 times, and the final classification accuracy was averaged across these 25 runs. We evaluated average classification accuracy, standard deviation, sensitivity and specificity of the classifier for all the feature types. Sensitivity and specificity are statistical measures to evaluate the class-wise performance of the classifier. Here, the sensitivity or the true positive rate referred to the accuracy of classifying face trials to *Face* class, i.e., the percentage of face trials that were correctly identified as face class, and specificity or the true negative rate referred to the proportion of no-face trials that were correctly identified as the *No-face* class.

In this work, we adopted a data-driven approach to investigate the role of prestimulus activity in face pareidolia. This approach resulted in a huge number of features considering the dimensions of frequency, time and channels. Many of these features are redundant and irrelevant for the problem at hand. Feature selection procedures are effective in dimensionality reduction, eliminating irrelevant features, improving learning accuracy and increasing result comprehensibility. However, in multivariate pattern analysis (MVPA) studies of neuroscience there is usually a huge imbalance between the number of features and samples. To avoid possible overfitting due to this, the feature selection was performed only on the training set while evaluating the performance of model (both feature selection and classifier) on unseen test data. The observed classification accuracy was reasonably good, suggesting the relevance of features for discriminating the two classes. Also, the problem here can be compared to the feature selection problem in micro-array data [51, 52], where the number of features far exceeds the observations and univariate methods such as *t* test are widely popular.

## Results

### Subject-wise analysis

The analysis of TFPS64, TFPSL, TFPSR and DATFPS features was performed for each participant. TFPS64 feature was chosen from all 64 scalp electrodes independent of participants. TFPSL, TFPSR and DATFPS considered left, right and the difference between left and right hemispheric electrodes, respectively, which included all scalp electrodes except 10 midline electrodes (Fpz, AFz, Fz, FCz, Cz, CPz, Pz, POz, Oz and Iz).

**Fig. 5:**
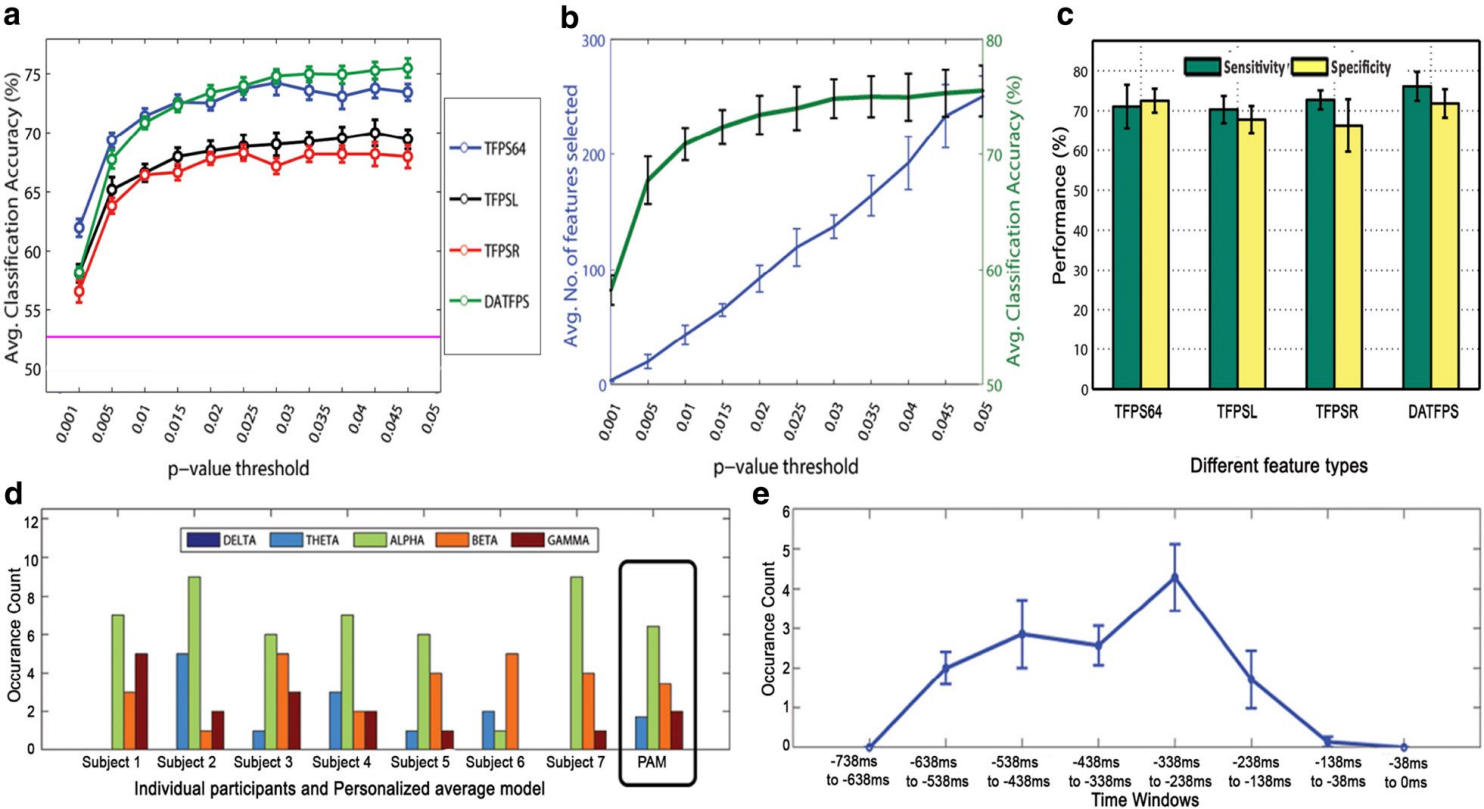
Results of subject-wise analysis: **a** Classification performance of different features with respect to different *p* value thresholds that used in feature selection method. Average classification accuracy of time–frequency power spectrum features of all 64 electrodes (TFPS64), left hemispheric electrodes (TFPSL), right hemispheric electrodes (TFPSR) and differential asymmetry between hemispheric features (DATFPS) are represented along with empirical chance level (pink horizontal line). Error bars indicate standard error of mean (SEM). **b** Representation of number of selected features and average classification accuracy of DATFPS feature with respect to different *p* value thresholds as DATFPS feature set yielded the best performance for all subjects. **c** Sensitivity and specificity performance (in %) for each feature type. Error bars indicate standard deviation (SE) across subjects. **d** Representation of occurrence count of dominant features. Band-wise dominant features for each subject is shown for DATFPS feature type. Among five EEG frequency bands, maximum selected features belonged from alpha frequency band. **e** Temporal course of occurrence count of dominant features. Error bars indicate SEM across subjects

Figure 5a shows the classification outcome. The average classification accuracy was plotted along with the empirical chance level around 54% [53] by varying the *p* value threshold from 0.001 to 0.05. We started with the *p* value smaller than a predefined threshold 0.001 and then selected the *p* value threshold of interval of 0.005 till the features with their *p* value smaller than 0.05. Here, we empirically determined a suitable threshold for selecting the features. Figure 5b shows that by increasing *p* value threshold of the *t* test the number of selected features increased and the classification accuracy tends to saturate (Fig. 5a). With a stricter threshold (for lower *p* values), the number of selected features decreased, but this might not be sufficient to discriminate between the two classes, as represented by the low classification accuracy. Hence, the *p* value was gradually increased to find the optimal threshold beyond which the classification accuracy did not show much improvement.

Table 2 shows the PAM classification performance of ANN classifier using these four feature types. We only picked optimal *p* values, of 0.025, 0.04, 0.025 and 0.035 for TFPS64, TFPSL, TFPSR and DATFPS, respectively. Additional file 1: Table A1 specifies the number of selected features for the above mentioned specific *p* values corresponding to feature types.

**Table 2:** Average classification accuracy (± standard deviation) for each feature type

Subject	Classification performance of individual subjects (in %)			
	TFPS64	TFPSL	TFPSR	DATFPS
	*p* value: 0.025	*p* value: 0.04	*p* value: 0.025	*p* value: 0.035
Subject1	$\mathit{74 }.\mathit{80 } \pm \mathit{6 }.\mathit{06 }$	$69.60 \pm 6.41$	$67.18 \pm 6.94$	$73.33 \pm 6.76$
Subject2	$74.45 \pm 6.63$	$70.89 \pm 7.20$	$68.60 \pm 6.84$	$\mathit{77 }.\mathit{24 } \pm \mathit{7 }.\mathit{38 }$
Subject3	$68.12 \pm 5.84$	$65.53 \pm 5.59$	$65.01 \pm 6.07$	$\mathit{73 }.\mathit{17 } \pm \mathit{6 }.\mathit{95 }$
Subject4	$74.59 \pm 6.23$	$73.64 \pm 5.90$	$67.44 \pm 6.09$	$\mathit{77 }.\mathit{32 }\pm \mathit{6 }.\mathit{59 }$
Subject5	$\mathit{73 }.\mathit{72 }\pm \mathit{6 }.\mathit{47 }$	$66.58 \pm 6.28$	$70.10 \pm 6.69$	$72.95 \pm 6.56$
Subject6	$\mathit{76 }.\mathit{64 } \pm \mathit{5 }.\mathit{80 }$	$66.76 \pm 6.40$	$69.18 \pm 6.14$	$76.16 \pm 6.30$
Subject7	$73.92 \pm 6.08$	$70.82 \pm 5.53$	$70.51 \pm 7.37$	$\mathit{74 }.\mathit{76 }\pm \mathit{6 }.\mathit{20 }$
PAM	$73.75 \pm 2.66$	$69.12 \pm 2.93$	$68.29 \pm 1.19$	$\mathit{74 }.\mathit{99 }\pm \mathit{1 }.\mathit{92 }$

*PAM* personalized average model, *TFPS64* time–frequency power spectrum of 64 electrodes (*p* < 0.025), *TFPSL* time–frequency power spectrum of left hemisphere (*p* < 0.04), *TFPSR* time–frequency power spectrum of right hemisphere (*p* < 0.025); *DATFPS* differential asymmetry of TFPS features (*p* < 0.035). These *p* values are uncorrected

For each subject, among four feature types, which yields highest performance are represented in italic form

Next, we studied the sensitivity and specificity (see Sect. 2.7) of our classifier model; Fig. 5c shows the findings for four feature types. We found that the sensitivity (accurately classifying *face* trials) and specificity (accurately classifying *no-face* trials) were comparable to the overall accuracy for these four feature types.

As individualized differences were expected with respect to the ability to perceive face pareidolia [25], we performed subject-dependent classification where models for each subject were trained separately. In this work, we report the individual as well as averaged classification performance in this framework, which is referred to as personalized average model. In general, experimental designs that involve personalized model analyses provide higher statistical power and therefore require fewer subjects compared to those that involve subject-independent analysis [27].

#### Feature usage

Here, we focused our analysis on identifying the features that were most consistent in discriminating between the two classes. Specifically, we were interested in identifying the critical frequency band(s) and time period(s). We performed this analysis with DATFPS features due to its better performance across participants. The classification framework employed random downsampling (25 times iteration) of the dataset with sixfold cross-validation. Thus, 150 $(25 \times 6)$ classification models were generated, and each model was constructed using different feature sets. In order to identify the consistent features, a histogram of occurrence of the features was created; a feature was considered to be consistent when that feature occurred over a threshold (at least 60% of maximum occurrence of features).

Figure 5d shows the band-wise distribution of the most consistent features selected at individual participant level. We found that the differential asymmetry in the alpha frequency band consistently emerged with the highest discriminating ability for all participants.

Similar to the dominant band identification, time localization analysis was also performed to identify a time period most critically involved in the prestimulus period predicting the perceptual decision in face pareidolia. Here, the whole 738 ms of prestimulus period was divided into eight windows: first seven windows, each of 100 ms duration, and the last window of 38 ms duration immediately prior to stimulus onset. The time windows where the number of occurrences of a feature exceeds a threshold (at least 60% of maximum occurrence of features) were considered as dominant or critically involved. Figure 5e shows the consistency of different time windows. We found that time windows from 538 to 238 ms before stimulus onset contained the features with better and consistently higher classification performance.

### Analysis of common feature set

In order to spatially localize the features, we considered common features across participants, and these were referred to as common feature set. The steps are illustrated next.

**Fig. 6:**
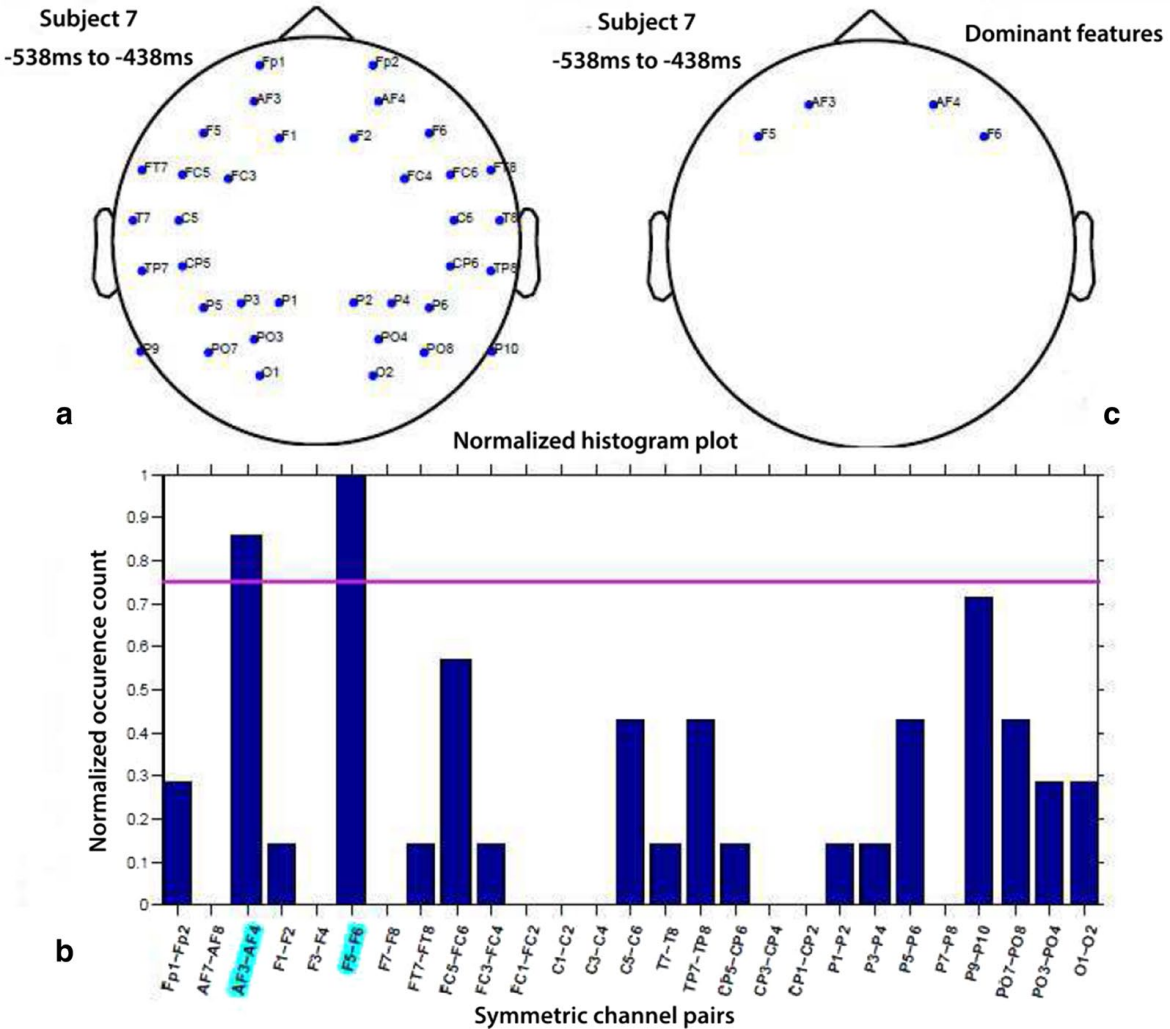
Steps of common feature analysis: **a** channel pairs selected at least once over all folds, **b** normalized histogram plot of channel pairs and **c** only dominant channel pairs

Step 1: Selection of dominant features in each participant (refer to Fig. 6)

- Different feature sets got selected in each fold of CV.
- Histogram plot describing occurrence of features was arrived at.
- A feature was considered dominant when its occurrence exceeds a threshold (here, 75% of the maximum occurrence of features).

Step 2: Computation of common feature set across subjects

**Fig. 7:**
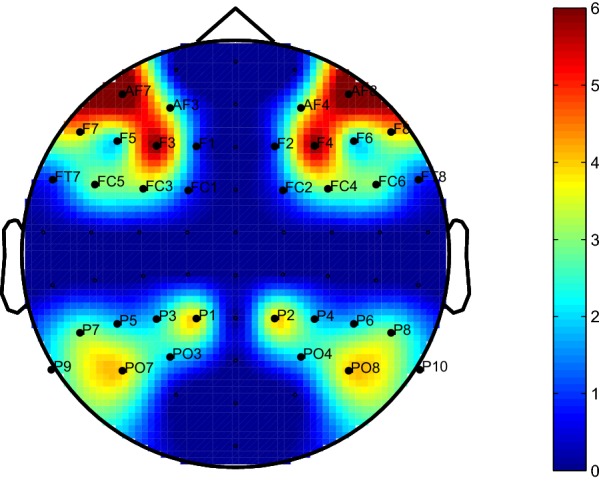
Commonality index: degree of commonality of each electrode for dominant features. The degree of use was color coded, according to the color bar on the right (as the spectral differences were derived from symmetric pairs, the symmetric patterns were formed)

- For any given feature, commonality index ($C_{\mathrm{i}}$) was calculated as the number of subjects for which it was found dominant. It can take values between 0 and 7, i.e., $0 \le {C_{\mathrm{i}}} \le 7$.
- Dominant features with $C_{\mathrm{i}} \ge$ 5 were considered as common feature set across subjects since this threshold approximately represents the 70% of the subjects. If threshold was increased to 6 (i.e., above 85%), the computation of common feature set across participants become more strict. It was observed that this results in selection of four features, from which it is difficult to infer the spatial pattern. Further it was observed that for threshold = 7 no features are found common in all subjects. However, if we decrease threshold $\le \,4$, almost all features were found common across the subjects. Hence, we chose the threshold of 5 as a reasonable indicator of the consistency of brain responses across subjects. For example, Fig. 7 shows the commonality index of corresponding electrodes for DATFPS features, where each feature is associated with an electrode pair (left–right).

In Fig. 7, we plotted the commonality index for each feature at both associated electrodes in the left and right hemispheres. Hence, the plot is perceived as symmetry between both hemispheres.

Features were extracted as explained in Sect. 2.5, from the electrode positions found in the common feature set. The individual TFPS of these 39 electrodes is named as TFPS39 (17 pair electrodes and AFz, Fz, FCz, POz, Pz). Similarly, the hemispheric features are labeled as TFPSL17 and TFPSR17 as there are 17 symmetric electrode pairs in that commonly targeted zone and DATFPS17 are the differential asymmetry of these 17 electrode pairs.

The data of each participant were analyzed with the common feature set using the same classification framework as discussed earlier. Though average classification accuracy was calculated by varying the *p* value threshold from 0.001 to 0.05 in *t* test feature selection for each of TFPS39, TFPSL17, TFPSR17 and DATFPS17 feature types, we only showed the classification performance in those *p* value thresholds where the accuracy was high and consistent. The levels of threshold at saturation points were 0.035, 0.03, 0.035 and 0.045 in the case of TFPS39, TFPSL17, TFPSR17 and DATFPS17, respectively. Table 3 indicates the average classification performance of the common feature set. The number of selected attributes for the above-mentioned specific *p* values corresponding to the TFPS39, TFPSL17, TFPSR17 and DATFPS17 feature types is displayed in Additional file 1: Table A2.

**Table 3:** Average classification accuracy (± standard deviation) of common feature set

Subject	Classification performance of individual subjects (in %)			
	TFPS39	TFPSL17	TFPSR17	DATFPS17
	*p* value: 0.035	*p* value: 0.03	*p* value: 0.035	*p* value: 0.045
Subject1	$69.98 \pm 6.11$	$66.92 \pm 6.70$	$63.80 \pm 6.19$	$\mathit{70 }.\mathit{05 } \pm \mathit{7 }.\mathit{16 }$
Subject2	$73.08 \pm 6.27$	$70.38 \pm 6.73$	$67.74 \pm 6.84$	$\mathit{73 }.\mathit{30 } \pm \mathit{6 }.\mathit{26 }$
Subject3	$\mathit{70 }.\mathit{67 } \pm \mathit{6 }.\mathit{57 }$	$65.81 \pm 6.56$	$62.69 \pm 5.45$	$69.74 \pm 6.76$
Subject4	$72.67 \pm 5.72$	$69.43 \pm 7.13$	$65.62 \pm 6.30$	$\mathit{74 }.\mathit{33 } \pm \mathit{6 }.\mathit{52 }$
Subject5	$69.86 \pm 6.83$	$65.58 \pm 6.84$	$68.17 \pm 6.76$	$\mathit{71 }.\mathit{03 } \pm \mathit{7 }.\mathit{16 }$
Subject6	$\mathit{75 }.\mathit{04 } \pm \mathit{5 }.\mathit{83 }$	$67.86 \pm 6.41$	$69.43 \pm 6.93$	$72.82 \pm 6.38$
Subject7	$71.93 \pm 6.24$	$65.92 \pm 6.07$	$68.17 \pm 6.34$	$\mathit{72 }.\mathit{92 } \pm \mathit{6 }.\mathit{56 }$
PAM	$71.89 \pm 1.88$	$67.41 \pm 1.89$	$66.52 \pm 2.53$	$\mathit{72 }.\mathit{03 } \pm \mathit{1 }.\mathit{76 }$

*PAM* personalized average model, *TFPS39* time–frequency power spectrum of 39 electrodes from common feature set (*p* < 0.035), *TFPSL17* time–frequency power spectrum of 17 electrodes from left hemisphere (*p* < 0.03), *TFPSR17* time–frequency power spectrum of 17 electrodes from right hemisphere (*p* < 0.035), *DATFPS17* differential asymmetry of TFPS of 17 electrode pairs (*p* < 0.045). These *p* values are uncorrected

For each subject, among four feature types, which yields highest performance are represented in italic form

**Fig. 8:**
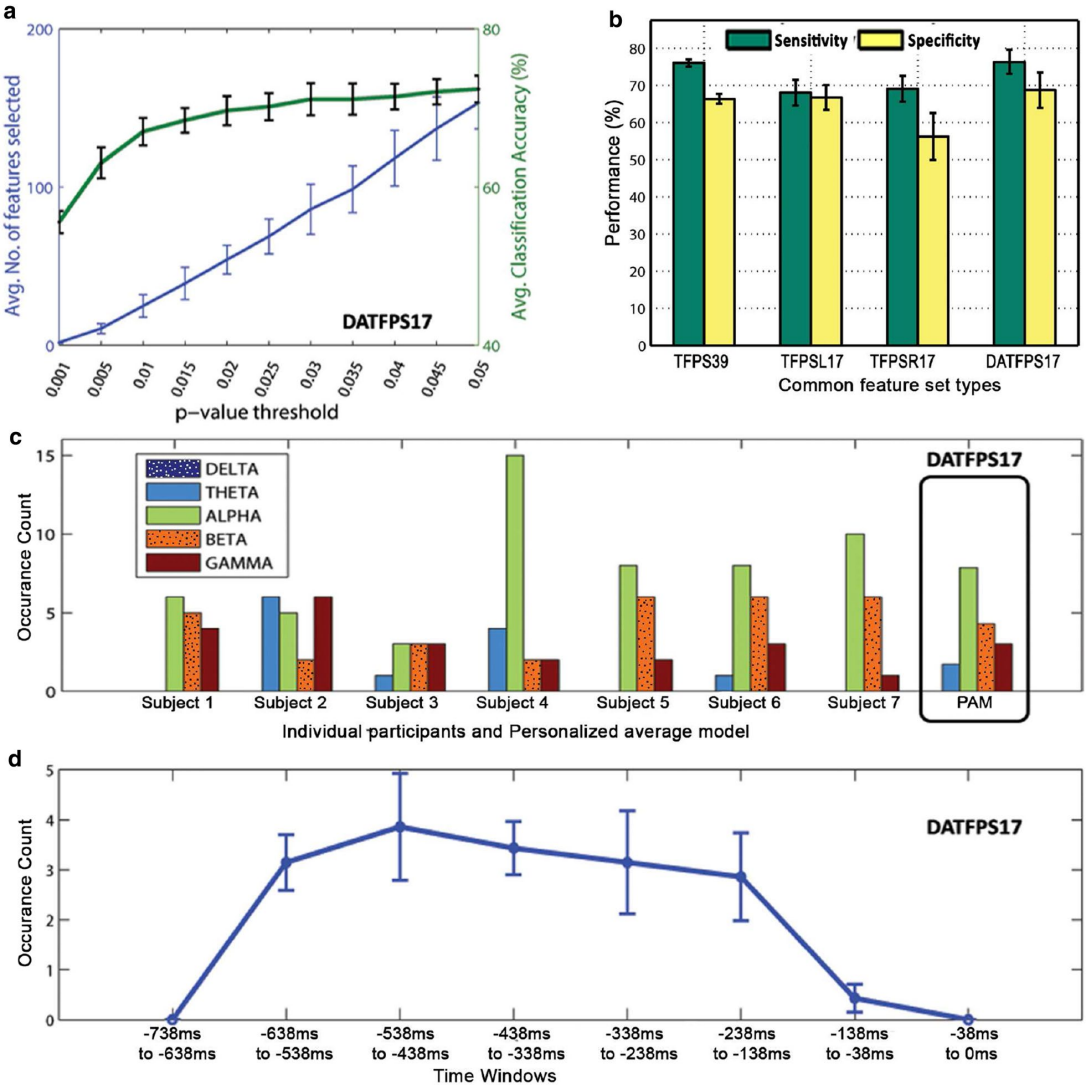
Results of common feature set analysis: **a** Number of selected features and average classification accuracy are shown for hemispheric asymmetry features (DATFPS17) with respect to different *p* value thresholds as DATFPS17 feature set yielded the best accuracy among all common feature sets. **b** Grouped sensitivity and specificity performance (in %) are shown in bar plots with error bars that indicate standard deviation (SE) along all subjects. **c** Presentation of occurrence count of dominant features. Band-wise dominant features for each subject is shown for DATFPS17 features type. Among five EEG frequency bands, maximum selected features belonged from alpha frequency band. **d** Temporal course of occurrence count of dominant features. Error bars indicate SEM along all subjects

Table 3 shows that the averaged classification performance of ANN classifier using DATFPS17 was distinctly better among all four feature types (TFPS39, TFPSL17, TFPSR17 and DATFPS17) across all subjects with maximum classification accuracy of $72.03 \pm 1.76$%. Figure 8a represents the number of selected features and average classification accuracy of DATFPS17 on different thresholds; by increasing the *p* value, the classification accuracy tended to saturate, but the number of selected features increased.

In order to characterize classifier performance, we analyzed sensitivity and specificity measures of the classifier on the set of common features similarly as done before for TFPS64, TFPSL, TFPSR and DATFPS feature types. Bars in Fig. 8b show the sensitivity of our classifier was comparable the specificity for all feature types.

#### Feature usage

Dominant frequency band identification was analyzed for the set of common features similarly as done earlier (Fig. 8c). Again, we observed that the alpha was the most dominant frequency band from where maximum features were chosen. Following the same procedure, as followed in the case of DATFPS feature type, time localization analysis was done on the set of common features only on DATFPS17 features. We observed that the most dominant features were found within $-\,638$ ms to $-\,238$ ms (Fig. 8d).

### Moving window analysis

In order to get an idea of the timing window over which better classification performance occurs, we performed a moving window analysis with different window sizes. To consider the window size which in turn gives the best time resolution, we took note of the following. For complex Morlet wavelets, the time resolution at a particular wavelet scale was computed $\sigma _{\mathrm{t}} = \frac{n}{2\pi f_{\mathrm{c}}}$, where $f_{\mathrm{c}}$ is the center frequency and parameter *n* denotes the number of cycles (in this study, n = 4 [35]). This equation defines the trade-off between temporal precision and frequency, i.e., higher frequencies (beta and gamma bands) can be well resolved in time, whereas low frequencies need wider wavelets. For delta band ($f_{\mathrm{c}} = 2.6$ Hz), $\sigma _{\mathrm{t}} = 244.8$ ms, which constrained us to use window size around this value. Hence, we fixed the highest time resolution to 246 ms to make the computation of wavelet features possible in all bands. In addition, the value of 246 ms allowed the exact division of prestimulus period into integer number of windows. The other window sizes considered are 369 ms, 492 ms and 615 ms which is in arithmetic progression of 123 ms, half of 246 ms. The next in sequence was 739 ms which covers the entire prestimulus period and considered in other part of the paper. We considered 123 ms shift of time window to obtain the time profile.

For each considered window size, the window was further partitioned into consecutive 10 ms segments and the mean power of each wavelet band in these segments was used as features. These features carried information localized in time, and the numbers of features were higher for longer window lengths. The classification framework was used with the DATFPS17 features. The results, shown in this section, considered *p* value threshold of 0.05 for the *t* test.

**Fig. 9:**
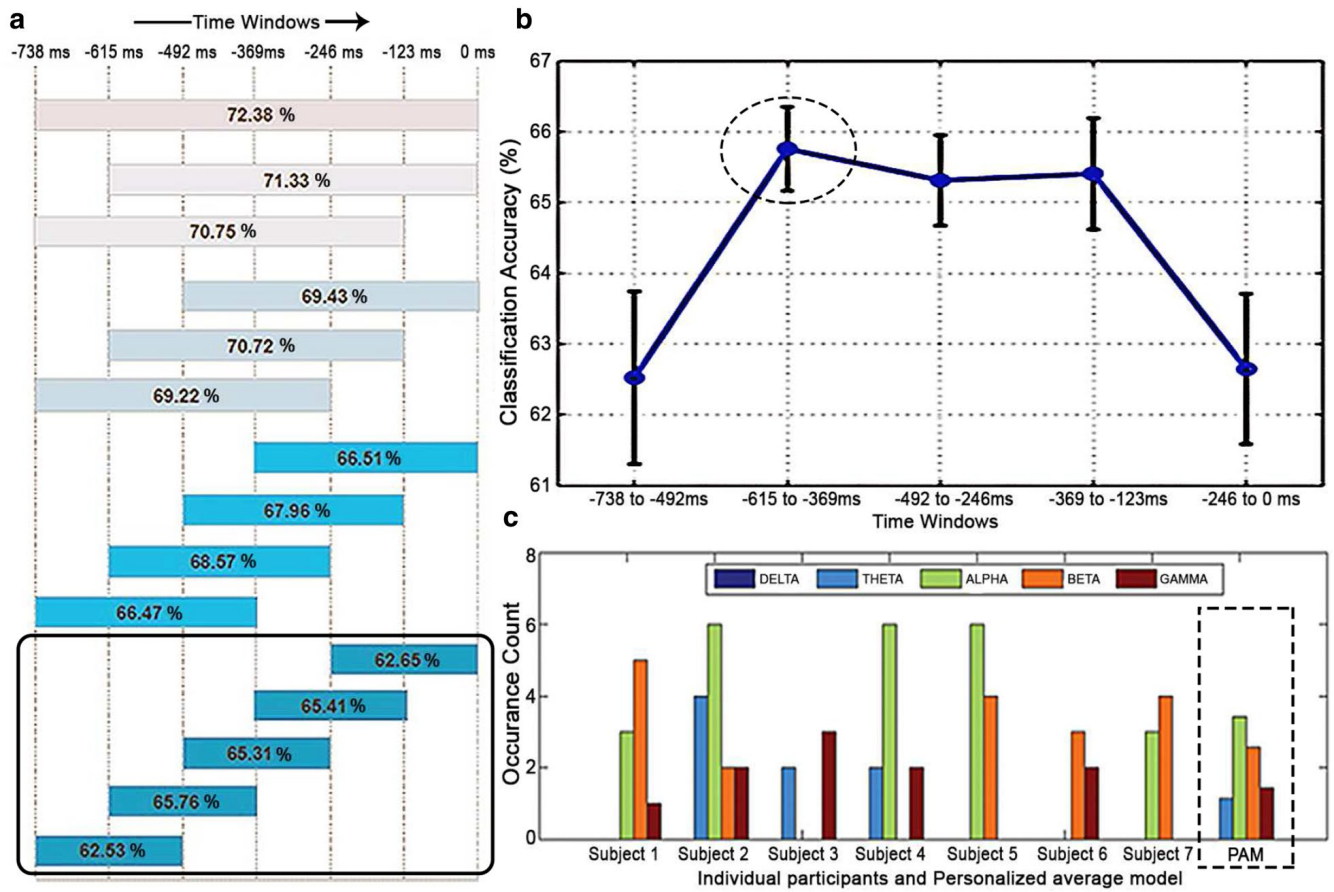
Results of moving window analysis: **a** Arrangement for moving windows along with classification accuracy averaged over all subjects using DATFPS17 feature. **b** Error bar indicates SEM of individual subjects accuracies in this feature type over each moving window of 246 ms. Features of $-\,615$ ms to $-\,369$ ms window yielded the highest accuracy. **c** For this time window, band-wise occurrence count of dominant features for each subject and PAM using DATFPS17 feature type is shown

The arrangement for moving windows along with classification accuracy averaged over all participants is shown in Fig. 9a. The best classification accuracy was $72.38 \pm 1.84\%$ corresponding to the window length equal to the whole prestimulus period. This could be because it captured the entire time and frequency information that was good to compute wavelet analysis-based features.

To localize time to the maximum possible extent, we selected window size of 246 ms which was minimum for the computation of wavelet features. We observed (Fig. 9b) that the average classification performance showed an increasing trend up to the middle 246 ms window ($-\,615$ ms to $-\,369$ ms), and then, it followed a decreasing trend. Specifically, the time period $-\,615$ ms to $-\,369$ ms showed the most discriminative power with DATFPS17 features. Interestingly, the time period immediately before the stimulus onset was associated with lower classification accuracy. Overall, we found that it was possible to predict the perceptual decision in face pareidolia using prestimulus brain activity across various time windows with maximal accuracy around 500 ms before the stimulus onset.

Finally, we were interested in finding the frequency band specificity on that specific time window of each participant corresponding to the maximum classification accuracy. Figure 9c shows that maximum selected features indeed belonged to the alpha frequency band.

## Discussion

The present study investigated whether prestimulus brain oscillations could systematically predict post-stimulus perceptual decision in a face pareidolia task on a trial-by-trial basis. Using a pattern classification approach for large-scale EEG signals, we found that it is indeed feasible to predict the perceptual decision considerably higher than chance level based on the prestimulus activity alone. Further, the perceptual decision information was specifically coded in the prestimulus alpha oscillations and in the asymmetric distribution of oscillatory features between the two hemispheres.

Prestimulus brain activity shapes the post-stimulus perception: This study inspected the causal impact of prior expectation before the stimulus onset on the post-stimulus perception in face pareidolia. Participants were presented with noise images, but prior information on the faces being hidden in these images led to the participants reporting seeing faces on many trials. We demonstrated that it was possible to capture features of large-scale ongoing brain activities prior to the presentation of stimuli that could reliably predict the participants responses, *face* or *no-face*, on trial-by-trial basis. Our classifier model produced a mean accuracy around 75% that was substantially above the chance level around 54% [53]. This finding is consistent with a growing body of the literature establishing the existence of neural signals that predetermine perceptual decisions [10, 11, 13, 15–17, 54]. It is known that any decision made in the post-stimulus period is not entirely dependent on the stimulus alone; instead it relies on several top-down processes including expectations, prior knowledge and goals, formed in the prestimulus period [55]. This predictive impact of prestimulus brain activity may offer potential advantage in enhanced preparedness in avoiding aversive situation [56]. Several studies also investigated the neurophysiological mechanisms underlying prestimulus processing. For example, fMRI studies have revealed predictive signals in the hippocampus [57, 58]. Hindy et al. [57] found that memory-based expectations in human visual cortex are related to the hippocampal mechanism of pattern completion. The study [59] reported anticipatory firing to expected stimuli in the medial temporal lobe, including the hippocampus. A prior study [60] showed the channels corresponding to the maximal coefficients of spatial pattern vectors may be the channels most correlated with the task-specific sources, i.e., frontal and parieto-occipital regions activate for ‘face’ and ‘no-face’ imagery class, respectively. These findings suggest a mechanism of how prior expectations in the prestimulus period may affect post-stimulus decision making.

Further, [12] had suggested that neural signals present before stimulation can bias decisions at multiple levels of representation when evaluating stimuli. In this study, since the participants were instructed that face was present in some of the trials, the prestimulus phase is associated with anticipatory processing. According to [2], this phase could involve both expectation and attention facilitating top-down processing, which in turn affects the perceptual decisions. While expectation facilitates interpretation of the stimulus and detection of objects that are likely to be present in the visual environment, attention alleviates computational burden by prioritizing sensory inputs according to their salience or relevance to current goals [61]. In our study, the prior expectation manipulates the perception of participants affecting their performance. On the other hand, attention may facilitate the participants to recall face templates from memory and identify face-like features in the upcoming white noise images via top-down processing. Interestingly, in our study, we observed large variations across our participants in terms of the prestimulus features predicting *face* or *no-face* decision, yet the features were quite stable within an individual, and further, we could still identify a set of common feature set in the prestimulus period. We did observe a wide fluctuation, from 1:3 to 1:1, in *face* to *no-face* trial ratio, but such individual differences in face pareidolia had not been systematically investigated yet. One possible reason for the variability in perceptual performance across participants is likely to stem from the participants attention capabilities that should be reflected in ongoing oscillatory activity, already present before stimulus presentation [62].

Hemispheric differential asymmetry features yield the best classification performance and capture the prior influence well: Identifying the essence of differences between the left and right hemisphere of the brain is a key component of understanding functional organization of neural processing [63]. Hence, we analyzed differential hemispheric asymmetry features on a single-trial basis. Despite large inter-individual differences in the involvement of various brain regions during the formation of expectation in the prestimulus period, our classifier demonstrated that the neural signature at the hemispheric level was largely consistent across participants, and further, the hemispheric asymmetry was causally linked to the perceptual decision. It is widely believed that the advantages of hemispheric asymmetries originated in more efficient cognitive and affective processing; hence, it is often implied that the relationship between hemispheric asymmetry and cognitive performance is linearly positive: The higher the degree of lateralization in a specific cognitive domain (here anticipation), the better the performance in corresponding task [64, 65]. Taken together, our research utilized conscious anticipation [66] to assess contralateral hemispheric differences for prestimulus expectation in face pareidolia visual perception.

Current cognitive neuroscience models predict a right hemispheric dominance for face processing in humans. However, neuroimaging and electromagnetic data in the literature provide conflicting evidence of a right-sided brain asymmetry for decoding the structural properties of faces. Interestingly, the fMRI-based study in [67] showed an activation of fusisorm face area (FFA) only in the right hemisphere in about half the subjects (both men and women), whereas the other subjects showed bilateral activation. These results raised the possibility of functional hemispheric asymmetry in the FFA. Studies addressing this possibility have provided conflicting evidence, where [68–72] found stronger activity in the right hemisphere, while other studies failed to support the notion of a strict right lateralization (e.g., [73] performed in five men and seven women). The study in [74] found significantly higher fMRI responses to faces than to objects in both the left and right mid-fusiform gyrus regions, although this effect was slightly greater in the right than the left FFA. Another study in [63] provided important clues regarding the functional architecture of face processing, suggesting that the left hemisphere is involved in processing ‘low-level’ face semblance, and perhaps is a precursor to categorical ‘deep’ analyses on the right. Using single-trial EEG signal, our result of hemispheric asymmetry, lies on the same line as the neuroimaging study [67].

Ongoing oscillations in the alpha frequency range play a strong role in predicting the effect of prior expectation: Different frequency bands are related to various cognitive and perceptual processes [75, 76]. In our study, we found that the alpha band prestimulus oscillations were critically involved with the prediction of future decision. This result was in line with other studies demonstrating the causal role of alpha oscillations in the prestimulus period in shaping post-stimulus task processing. For example, the strength of prestimulus alpha power was associated with detecting near-threshold stimuli [77, 78]. It has been found that the perception of low-threshold somatosensory stimuli is related to high parietal alpha power [77]. Also, it has been established that conscious visual perception of a cue stimulus in an orienting shifting paradigm is related to high prestimulus power in the lower alpha frequency range (6–10 Hz) [78]. Several studies have reported that increased alpha oscillations reflect higher top-down processing [79, 80]. Many existing studies have established the relationship between ongoing oscillations in the alpha frequency range (around 8–13 Hz) and expectation processes [6, 7, 62]. In a recent work, it has been found that low-frequency alpha oscillations can serve as a mechanism to carry and test prior expectation about stimuli [81]. Our results extend these studies by demonstrating that the large-scale oscillatory features in the alpha band could be captured at the single-trial level that possess significant discrimination ability to influence future choice options.

Certainly, our study has some limitations. For example, we analyzed the EEG data at the sensor level; therefore, the spatial resolution of our findings was limited. A better localization of prestimulus brain activity to predetermine perceptual decisions could be performed by reconstructing the neural sources on trial-by-trial basis. However, individual magnetic resonance image (MRI) is required for an accurate source reconstruction, which was not available in our study. This study involved young adults with six women among seven participants. In an ERP study of face pareidolia, it was noted before that women perform better at seeing faces where there are none [25]. Hence, the findings of this study cannot be generalized across gender. Future studies can be carried out by considering subjects from all age groups and equal participation from both genders. Further, we focused our analysis only on the neural oscillations, and future research can explore the potential contribution of connectivity measures as suitable features for classification at the structural brain level. Thus, the future scope of this work would be to analyze the prior expectation using different feature extraction techniques.

## Conclusion

Using an EEG-based decoding approach for face pareidolia, this study performed a systematic feature extraction followed by single-trial classification of brain signals. The aim was to investigate the influence of prior expectation in perceiving a face in a pure noise stimulus. We demonstrated that spatiotemporal spectral signatures in the prestimulus brain activity could significantly predict future decision, *face* or *no-face*, on a trial-by-trial basis. The neural signature at the hemispheric level was largely consistent across participants, and furthermore, we found that the alpha band prestimulus oscillations were critically involved in making the prediction of future decision. In summary, this study demonstrated the usefulness of machine learning techniques in predicting decisions from prior brain states on a single-trial basis.

## Additional file

**Additional file 1.** The average number of selected attributes for different feature types.
