# Supplementary material for: A machine learning approach to predict perceptual decisions: an insight into face pareidolia
Source: Brain Inform. 2019 Feb 5;6(1):2. doi: 10.1186/s40708-019-0094-5 (PMC6363645; doi:10.1186/s40708-019-0094-5)
Supplement: Supplementary file 1 — Additional file 1. The average number of selected attributes for different feature types. [file 40708_2019_94_MOESM1_ESM.pdf]

Supplementary Information

Table A1: Average number of selected features for each feature types

Subject	Number of features of individual subjects			
	**TFPS64**	**TFPSL**	**TFPSR**	**DATFPS**
	p-value:0.025	p-value:0.04	p-value:0.025	p-value:0.035
Subject1	286	221	114	147
Subject2	271	202	115	171
Subject3	347	286	95	184
Subject4	351	234	131	167
Subject5	230	144	103	134
Subject6	316	179	216	159
Subject7	369	255	169	189
PAM	310	217	135	**164**

Note: PAM = Personalized average model. TFPS64 = Time-frequency power spectrum of 64 electrodes ($p < 0.025$); TFPSL = Time-frequency power spectrum of left hemisphere ($p < 0.04$); TFPSR = Time-frequency power spectrum of right hemisphere ($p < 0.025$); DATFPS = Differential asymmetry of TFPS features ($p < 0.035$). These p-values are uncorrected.

Table A2: Average number of selected features of common feature set

Subject	Number of features of individual subjects			
	**TFPS39**	**TFPSL17**	**TFPSR17**	**DATFPS17**
	p-value:0.035	p-value:0.03	p-value:0.035	p-value:0.045
Subject1	291	120	128	132
Subject2	290	101	114	134
Subject3	194	102	102	109
Subject4	308	111	117	173
Subject5	224	84	91	124
Subject6	263	82	171	128
Subject7	382	139	169	158
PAM	279	106	127	**137**

Note: PAM = Personalized average model. TFPS39 = Time-frequency power spectrum of 39 electrodes from common feature set ($p < 0.035$); TFPSL17 = Time-frequency power spectrum of 17 electrodes from left hemisphere ($p < 0.03$); TFPSR17 = Time-frequency power spectrum of 17 electrodes from right hemisphere ($p < 0.035$); DATFPS17 = Differential asymmetry of TFPS of 17 electrode pairs ($p < 0.045$). These p-values are uncorrected.
